# Factors Associated With Health Care Utilization Among Transgender Adults in Brazil

**DOI:** 10.1111/jphd.70050

**Published:** 2026-03-03

**Authors:** Thiago Caldeira Diniz, Luciana Gravito de Azevedo Branco, Edu Turte Cavadinha, Ana Valéria Machado Mendonça, Maria Fátima de Sousa, Saul Martins Paiva, Fabiana Vargas Ferreira, Andreia Maria Araújo Drummond, Flavio Freitas Mattos

**Affiliations:** ^1^ Department of Social and Preventive Dentistry, School of Dentistry Federal University of Minas Gerais Belo Horizonte Minas Gerais Brazil; ^2^ Department of Collective Health, School of Health University of Brasília Brasília Federal District Brazil; ^3^ Department of Child and Adolescent Health, School of Dentistry Federal University of Minas Gerais Belo Horizonte Minas Gerais Brazil

**Keywords:** health care systems [Brazil], health services for transgender persons, sexual and gender minorities, transgender persons

## Abstract

**Objective:**

This study investigated the utilization of medical and dental services by transgender Brazilians and their associated factors.

**Methods:**

A national cross‐sectional survey was conducted in 2021 with 549 self‐identified transgender adults (231 male, 183 female, and 135 other gender) aged 18–61 years. Participants were recruited through social media using snowball sampling and answered an electronic structured questionnaire. Outcomes were the frequency of medical and dental appointments. Associated factors included sociodemographic characteristics, use of social name, partnership, children, religion, health information sources, perceived health needs, experience of gender‐based violence, and type of health services used. Odds ratios (OR) with 95% confidence intervals (CIS) were estimated using backward stepwise logistic regression models.

**Results:**

Respondents who attended public schools (OR 1.73; 95% CI 1.01–2.88), had no income (OR 1.96; 95% CI 1.01–3.85), and relied solely on the public health system (OR 2.48; 95% CI 1.36–4.50) were more likely to have semiannual medical consultations. Conversely, those who attended public schools (OR 1.89; 95% CI 1.09–3.31), had ≤ 9 years of schooling (OR 1.74; 95% CI 1.13–2.68), were unemployed (OR 1.88; 95% CI 1.04–3.42), or had experienced gender‐based violence (OR 1.57; 95% CI 1.02–2.41) were more likely to rarely or never attend dental consultations.

**Conclusions:**

Transgender Brazilians predominantly used the public health system, attending medical consultations semiannually but rarely dental appointments. Lower education, unemployment, lack of income, and exposure to gender‐based violence shaped healthcare utilization, highlighting persistent barriers to equitable and comprehensive health access.

## 1 Introduction

Within sexual and gender minority populations, transgender people face some of the greatest health disparities [1, 2, 3]. Multiple barriers to accessing education, employment, leisure, culture, and especially healthcare make them more susceptible to the development of chronic diseases and sexually transmitted infections (STIs) [4, 5, 6]. Human immunodeficiency virus (HIV) infection and acquired immunodeficiency syndrome (AIDS) [7], and mental disorders among transgender populations [8, 9, 10]. Discrimination and prejudice create health inequities and are determinants of illness and suffering in this population [4].

The Brazilian public health system, known as the Unified Health System (SUS), is a publicly funded, universal, and decentralized system that provides free access to comprehensive health care and includes actions to promote, protect, and restore health [11, 12, 13]. Brazil established in 2013 the National Policy for Integral Health Care for Lesbians, Gays, Bisexuals, Transvestites, and Transsexuals [4]. It regulates actions aimed at promoting health, preventing diseases, and providing good quality health services. It secures medical care for the transsexualization process, seeks to eliminate institutional prejudice, encourages the production of knowledge, and strengthens the representation of this social group in the development of SUS [4].

Although some progress has been made, barriers to healthcare utilization by transgender individuals remain significant. They may include sociodemographic inequalities, structural barriers, and experiences of discrimination which act as determinants of healthcare inequalities, and little is known about them among Brazilian transgender people. The hypothesis of this study is that sociodemographic vulnerabilities (such as lower education, unemployment, and income inequality), in combination with experiences of discrimination and low reliance on public health services, are associated with reduced and unequal use of medical and dental services among transgender adults in Brazil.

Inequalities and barriers faced by Brazilian transgender people reflect a broader Latin American and global pattern of exclusion. Brazilian studies describe discrimination within health services, non‐use of social names, lack of trained providers, and structural limitations of the SUS, which are associated with mental distress, exposure to violence, and avoidance of care [10, 11, 14]. The use of a social name is defined as the adoption and recognition of a self‐identified name that differs from the individual's legal name and is commonly used by transgender people in Brazil to affirm their gender identity in social and institutional contexts, including healthcare services.

Similar dynamics have been reported among transgender and nonbinary populations in other Latin American countries, such as Chile and Peru, where high unmet demand for gender‐affirming, mental, and basic health care persists due to long waiting times, financial constraints, and pervasive stigma [15, 16]. Regional reviews from Latin America and the Caribbean further highlight cisnormative professional training, limited community participation, and the absence or weakness of gender‐affirming policies as key programmatic barriers [17, 18].

This study aimed to characterize the adult transgender population in Brazil and to identify their use of medical and dental health services. The study also searched for associated factors that act as facilitators or barriers to the constitutional right of Brazilian transgender individuals to access comprehensive health care.

## 2 Methods

### 2.1 Procedures

This study was conducted in 2021 as part of the “Multicenter Study on the Socioeconomic, Geographic, Cultural, and Vulnerability Profiles of Transvestites and Transexual Individuals,” coordinated by the Center for Public Health Studies at the University of Brasília, with the participation of public universities across Brazil. The research project was approved by the Research Ethics Committee of the Faculty of Health Sciences at University of Brasília under CAAE 33798320.8.0000.0030.

The methodology was quantitative, cross‐sectional, and used an electronic questionnaire. The snowball sampling method, commonly used in hard‐to‐reach populations, was adopted to reach the sample size of 549 participants. This sampling strategy is particularly appropriate for transgender populations, who often experience social marginalization and mistrust of institutions, making them difficult to reach through conventional probability‐based sampling methods. Snowball sampling has been widely used in national and international studies investigating health, access to care, and social determinants among transgender and gender‐diverse populations [19, 20].

### 2.2 Participants

Knowledge of the number of transgender (transsexual or transvestite) individuals in Brazil is impaired by the lack of official data. In this study, the term transgender is used as an umbrella concept to describe individuals whose gender identity differs from their sex assigned at birth, including transsexual and transvestite identities, in accordance with established academic definitions [21]. The term “transvestite” is used in this study to reflect a specific socio‐cultural identity historically recognized in the Brazilian context and is not intended to carry a pejorative meaning. Its use follows established Brazilian academic and policy frameworks and aims to respect participants' self‐identification.

It is estimated that around 0.4% of the Brazilian population are self‐identified as transexual or transvestite, which would amount to approximately 850,000 people [14, 22]. To ensure reliability and representativeness of the information to be collected and analyzed, study sample design and calculation was scaled proportionally to the size of each Brazilian regional population. The procedure for proportional scaling was not fully randomized, and it was adopted as an approximation of representativeness. The sample size of 549 individuals was determined considering a finite population correction, a sampling error of ±10%, and a 90% confidence level.

Initial participants were identified through publicly available profiles of transgender activists, influencers, and community organizations on social media platforms, who shared the study invitation within their networks. These initial respondents constituted a small convenience‐based seed group, from which additional participants were recruited through peer referral. Although the exact number of participants recruited by each initial respondent was not systematically tracked, recruitment occurred through multiple referral waves until the target sample size was reached. Data collection took place over a defined period in 2021 and was concluded once the planned sample size of 549 respondents was achieved.

Respondents were reached through pages and profiles of influencers and transgender associations, direct messages on Instagram and Twitter (now X), and WhatsApp groups. Those who showed interest through social media were briefed about the research and provided with a link to access the informed consent form and the questionnaire. Individuals self‐identified as transgender, aged 18 or older, residing in Brazilian municipalities with more than 100,000 inhabitants, and who signed and returned the informed consent form were included in the sample until 549 respondents were reached. To minimize duplication of responses, each participant could only access the questionnaire once from the same IP address, and duplicate entries with identical sociodemographic profiles were checked and excluded. Completion of all questionnaire items was required for submission, and therefore no missing data were present for the variables included in the analyses.

### 2.3 Measures

The electronic questionnaire had 20 questions, divided into five topics which addressed: (1) personal identification, (2) education and professional training, (3) employment and income, (4) use of health services, and (5) human and social rights and security (Supporting Information Appendix A). The questionnaire was specifically developed for this multicenter study based on its research objectives and prior national investigations addressing transgender health and access to healthcare services. No items were directly adapted from previously validated instruments, and no formal psychometric validation or reliability testing was conducted. The questionnaire was not pilot tested prior to data collection, which may have influenced measurement properties and is acknowledged as a limitation of the study.

### 2.4 Grouping and Categorization Variables

The frequency of medical and dental services use by the participants was collected and analyzed as the outcome variables of the study. The frequency of medical appointments was dichotomized as up to semiannually and more than semiannually, with semiannual or more frequent consultations defined as the event of interest. The frequency of dental appointments was categorized as regularly versus never, rarely, or only in emergency situations, with infrequent use of dental services defined as the event of interest.

### 2.5 Grouping and Categorization of Associated Variables

Associated sociodemographic data that could act as barriers or facilitators for the study outcomes were also collected and analyzed after grouping and categorization. These variables were selected based on prior national and international literature on healthcare access among transgender populations and on conceptual frameworks addressing social determinants of health, discrimination, and structural barriers to healthcare utilization [4, 10, 11, 14, 18]. All continuous variables were dichotomized based on the median after descriptive analysis, ensuring balanced categories for regression modeling. The choice of these cut‐off points allows comparability with national surveys and reflects public health relevance.

The variable age was dichotomized by the median into 18–25 and 26–61 years. The local of residence was categorized according to the geographical regions of Brazil. The self‐declared skin color was collected according to the Brazilian Institute of Geography and Statistics [23] as Indigenous, Yellow, Brown, or Black. Participants' gender was categorized as female, male, and others. The social name, presence of children and fixed partners were dichotomized as yes and no. Respondent's religion was categorized as Christian, African, others, and none. The type of school attended was categorized as public, private, and both, and their educational attainment was dichotomized into up to 9 years (the number of years needed to complete national elementary education) and 10 or more years of schooling. Respondents' occupation was categorized as working and not working, and their monthly income was categorized as no income, up to $200.00, and more than $200.00. Their access to government financial assistance (social benefits) was dichotomized as yes and no. Participants' source of health information was categorized as digital, non‐digital, and both. Their self‐reported presence of mental health needs, previous experience of gender‐based violence, and the presence of chronic illnesses were dichotomized as yes and no. The kind of health services accessed by the participants was categorized as public, private/insurance, both, and none.

### 2.6 Data Analysis

Descriptive and multivariate analyses were conducted using SPSS version 21.0 (IBM Corp, Armonk, New York, United States). Multivariate analysis was performed through crude and adjusted Logistic Regression, using a backward stepwise model [24], to assess the association between exposure variables (age, skin color, gender, use of social name, partnership, children, religion, type of school attended, education level, occupation, income, government financial assistance, type of healthcare service used, and experience of gender‐based violence), and the outcome variables (frequency of medical and dental appointments). For medical consultations, the outcome modeled was having semiannual or more frequent medical appointments, with less frequent consultations used as the reference category. For dental consultations, the outcome modeled was rarely or never attending dental appointments or attending only in emergency situations, with regular dental attendance used as the reference category. The measure of association was odds ratio (OR) with 95% confidence interval (CI). Variables with *p* < 0.05 were considered statistically significant.

### 2.7 Reporting Guideline

This cross‐sectional study adheres to the STROBE statement for observational studies; the completed checklist is provided as Supplementary Information and is available from the corresponding author upon reasonable request.

## 3 Results

The descriptive analysis of the data is presented in Table 1. The respondents were between 18 and 61 years old and more frequently resided in the Southeast (39.2%) and Northeast (29.9%) regions of Brazil. There were similar proportions of respondents self‐identified as White (51.2%) and as Black, Brown, Indigenous, or Yellow (48.8%), according to the categories adopted in the analysis. Among the participants, 35.5% were unemployed, and 28.6% had no income, and 93.8% did not receive any type of government financial assistance.

**TABLE 1:** Descriptive analysis of sociodemographic data on the Brazilian transgender population and their utilization of health services.

Variables	*n*	%
Age (years)		
18–25	294	53.4
26–61	255	46.4
Residence		
Midwest	61	11.1
Northeast	164	29.9
North	59	10.7
Southeast	215	39.2
South	50	9.1
Skin color		
White	281	51.2
Indigenous, Yellow, Brown, or Black	268	48.8
Gender		
Female	183	33.3
Male	231	42.1
Other	135	24.6
Use of social name		
Yes	236	43.0
No	313	57.0
Children		
Yes	30	5.5
No	519	94.5
Partnership		
With a fixed partner	80	14.6
No fixed partner	443	80.7
Other	26	4.7
Religion		
Christian	96	17.5
African	99	18.0
Other	99	18.0
Non‐practicing	255	46.4
School type		
Public	235	42.8
Private	127	23.1
Both	187	34.1
Education		
Up to 9 years	208	37.8
10 or more years	341	62.2
Occupation		
Working	354	64.5
Not working	195	35.5
Monthly income		
No income	157	28.6
Up to $200.00	168	30.6
More than $200.00	224	40.8
Social benefit		
Yes	34	6.2
No	515	93.8
Health information		
Digital sources	82	14.9
Non‐digital sources	62	11.3
Both	405	73.8
Mental health needs		
Yes	228	41.5
No	321	58.5
Gender‐related violence		
Yes	357	65.0
No	192	35.0
Chronic illnesses		
Yes	178	32.4
No	371	67.6
Health services use		
Public	304	55.4
Private/Insurance	87	15.8
Both	142	25.9
None	16	2.9
Medical appointments		
Up to semiannually	335	61.0
More than semiannually	214	39.0
Dental appointments		
Rarely/never/in emergencies	310	56.5
Regularly	239	43.5

Regarding gender identity, participants predominantly identified as transgender women or transgender men, while approximately a quarter of the respondents reported gender identities outside the binary, which were grouped under the category ‘other genders’ for analytical purposes. Mostly, the respondents did not use a social name (57%) and did not have children (94.5%) or a fixed partner (80.7%). There was a significant proportion of respondents who had experienced gender‐based violence (65%). Out of the total sample, 67.6% did not have chronic diseases, and 61% attended medical consultations up to semiannually. However, 56.5% of the respondents rarely or never went to dental consultations or did so in emergencies only. The sole use of SUS for healthcare assistance was the most frequent (55.4%).

After adjusted analysis, respondents who attended only public schools (OR 1.73; 95% CI 1.01–2.88) had no income (OR 1.96; CI 1.01–3.85) and used solely public service (OR 2.48; CI 1.36–4.50) had higher chances of having semiannual medical consultations (Table 2).

**TABLE 2:** Analysis of the association between the characteristics of the Brazilian transgender population and their semiannual access to medical consultations.

Variable	Unadjusted model		*p*	Adjusted model		*p*
	OR	95% CI		OR	95% CI	
Age (years)^b^						
18–25	1.00			1.00		
26–61	0.84	0.60–1.19	0.344	0.76	0.53–1.11	0.166
Skin color^b^						
White	1.00			1.00		
Indigenous, Yellow, Brown or Black	1.38	0.98–1.95	0.066	1.28	0.87–1.87	0.206
Gender^b^						
Female	0.66	0.44–0.99	0.048	0.79	0.51–1.22	0.284
Male	1.00			1.00		
Other	1.38	0.88–2.16	0.161	1.52	0.92–2.50	0.100
Use of social name				^a^	^a^	^a^
Yes	1.00					
No	0.75	0.53–1.07	0.112			
Partnership				^a^	^a^	^a^
With a fixed partner	1.00					
No fixed partner	0.88	0.55–1.44	0.627			
Other	1.04	0.43–1.56	0.924			
Religion				^a^	^a^	^a^
Christian	1.00					
African	1.25	0.69–2.24	0.463			
Other	1.41	0.79–2.53	0.244			
None	1.35	0.83–2.22	0.226			
School type^b^						
Public	1.87	1.18–2.98	0.008	1.73	1.01–2.88	**0.048**
Private	1.00			1.00		
Both	1.81	1.12–2.93	0.016	1.55	0.89–2.69	0.122
Education				^a^	^a^	^a^
Up to 9 years	1.33	0.93–1.89	0.116			
10 or more years	1.00					
Occupation^b^						
Working	1.00			1.00		
Not working	1.14	0.80–1.63	0.466	0.72	0.40–1.30	0.276
Monthly income^b^						
No income	1.58	1.04–2.39	0.033	1.96	1.01–3.85	**0.049**
Up to US$ 200.00	1.23	0.82–1.87	0.318	1.10	0.70–1.74	0.673
More than US$ 200.00	1.00			1.00		
Social benefit^b^						
Yes	1.00			1.00		
No	1.02	0.70–1.48	0.918	1.26	0.83–1.92	0.274
Health information				^a^	^a^	^a^
Digital source	1.54	0.96–2.49	0.076			
Non‐digital source	1.15	0.66–1.98	0.619			
Both	1.00					
Mental health needs				^a^	^a^	^a^
Yes	0.88	0.62–1.25	0.880			
No	1.00					
Gender‐related violence				^a^	^a^	^a^
Yes						
No						
Chronic illnesses^b^						
No	1.00					
Yes	0.77	0.53–1.12	0.168	0.78	0.53–1.16	0.226
Health service use^b^						
Public	2.68	1.55–4.63	< 0.001	2.48	1.36–4.50	**0.003**
Private/Insurance	1.00			1.00		
Both	1.60	0.87–2.95	0.129	1.62	0.87–3.04	0.129

*Note*: Bold *p* values represent statistical significance.

Abbreviations: CI, confidence intervals; OR, odds ratio.

^a^ Variables not included in the final model—“Backward stepwise” (*p* < 0.05). Bursac et al. [15] 0.869.

^b^ Variables retained in the final adjusted model after backward stepwise selection were age, skin color, gender, school type, occupation, monthly income, social benefit, chronic illness, and health service use.

Also, after adjusted analysis, the odds of Brazilian transgender individuals to rarely or never attend dental consultations were higher among respondents who attended only public schools (OR 1.89; CI 1.09–3.31), had up to 9 years of schooling (OR 1.74; CI 1.13–2.68), were unemployed (OR 1.88; CI 1.04–3.42), or had experienced gender‐based violence (OR 1.57; CI 1.02–2.41) (Table 3).

**TABLE 3:** Analysis of the association between the characteristics of the Brazilian transgender population and their rare, never, or emergency access to dental consultations.

Variable	Raw model		*p*	Adjusted model		*p*
	OR	95% CI		OR	95% CI	
Age (years)						
18 to 25	1.00			1.00		
26 to 61	0.77	0.53–1.11	0.165	0.75	0.50–1.11	0.152
Skin color						
White	1.00			1.00		
Indigenous, yellow, brown or black	0.95	0.66–1.36	0.782	0.85	0.57–1.27	0.427
Gender						
Female	1.23	0.80–1.88	0.344	1.26	0.79–2.03	0.332
Male	1.00			1.00		
Other	1.37	0.85–2.23	0.196	1.07	0.60–1.86	0.798
Use of social name						
Yes	1.06	0.73–1.53	0.758	1.32	0.88–1.99	0.174
No	1.00			1.00		
Partnership						
With a fixed partner	1.00			1.00		
No fixed partner	1.19	0.70–2.03	0.515	1.27	0.71–2.26	0.426
Other	1.40	0.54–3.59	0.489	1.33	0.47–3.76	0.597
Religion				^a^	^a^	^a^
Christian	1.00					
African	1.12	0.59–2.11	0.725			
Other	1.23	0.66–2.31	0.509			
None	1.44	0.85–2.44	0.169			
School type						
Public	1.31	0.80–2.15	0.277	1.89	1.09–3.31	**0.025**
Private	1.00			1.00		
Both	1.77	1.07–2.92	0.026	1.18	0.65–2.17	0.581
Education						
Up to 9 years	1.63	1.12–2.36	0.010	1.74	1.13–2.68	**0.012**
10 or more years	1.00			1.00		
Occupation						
Working	1.00			1.00		
Not working	1.55	1.07–2.25	0.021	1.88	1.04–3.42	**0.038**
Monthly income						
No income	1.39	0.89–2.17	0.143	0.86	0.43–1.72	0.663
Up to US$ 200.00	1.37	0.88–2.11	0.161	1.03	0.63–1.68	0.915
More than US$ 200.00	1.00			1.00		
Social benefit				^a^	^a^	^a^
Yes	1.00					
No	0.86	0.58–1.27	0.439			
Health information				^a^	^a^	^a^
Digital source	0.97	0.58–1.63	0.911			
Non‐digital source	0.91	0.50–1.63	0.741			
Both	1.00					
Mental health need						
No	1.00			1.00		
Yes	1.14	0.79–1.65	0.474	1.11	0.73–1.71	0.618
Gender‐related violence						
Yes	1.60	1.08–2.38	0.019	1.57	1.02–2.41	**0.039**
No	1.00			1.00		
Chronic illnesses						
No	1.00			1.00		
Yes	0.83	0.56–1.22	0.344	0.75	0.49–1.15	0.192
Health service used						
Public	1.61	0.93–2.78	0.087	1.48	0.83–2.85	0.171
Private/Insurance	1.00			1.00		
Both	0.98	0.53–1.85	0.973	1.02	0.54–1.99	0.936

*Note*: Bold *p* values represent statistical significance.

Abbreviations: CI, confidence intervals; OR, odds ratio.

^a^ Variables not included in the final model—“Backward stepwise” (*p* < 0.05). Bursac et al. [15] 0.869.

## 4 Discussion

Ensuring healthcare services that are adaptable to the evolving needs of transgender individuals remains a key public health challenge, and the adoption of best practices is essential [13, 25]. Although this study does not aim to provide a representative sample of the Brazilian transgender population, the sociodemographic profile observed is consistent with patterns described in national studies and in regional literature from Latin America. Characteristics such as educational disadvantage, unemployment, income instability, and high exposure to violence have been repeatedly reported among transgender populations in Brazil and in neighboring countries, suggesting that the vulnerabilities identified here reflect broader structural trends rather than isolated features of the sample.

Brazil seeks to provide universal access to comprehensive health care for all its citizens through the SUS, a publicly funded and decentralized system grounded in the principles of universality, equity, and comprehensive care [11, 26]. Although a National Policy for Integral Health Care for Lesbians, Gays, Bisexuals, Transvestites, and Transsexuals has been established, important gaps remain between policy formulation and service delivery, particularly for transgender populations [4]. Structural limitations in healthcare infrastructure, insufficient professional training, and the uneven availability of gender‐affirming and oral health services continue to constrain access. In addition, the scarcity of population‐based data on transgender health and healthcare utilization hampers effective planning and evaluation of public policies. In this context, this study addresses a critical knowledge gap by providing one of the largest national studies to simultaneously examine medical and dental healthcare utilization among transgender adults in Brazil, contributing evidence that has been largely absent from both national and international public health dentistry literature.

A national survey on the access and the utilization of healthcare services among transgender individuals in the United States identified that this population delayed seeking care due to fear and past experiences of discrimination [9]. A recent scoping review analyzed the scientific literature on the oral health of LGBTQIAP+ individuals and recommended the encouragement of the production of scientific evidence that considers their treatment needs for public health planning [27].

Equity in healthcare access is fundamental for effective and fair healthcare systems and it is known that sociodemographic factors frequently play a significant role in determining who accesses healthcare services [9, 28, 29, 30, 31, 32]. In this study, transgender individuals who attended only public schools had a higher likelihood of consulting with doctors semiannually, when compared to those who attended only private schools. Respondents who attended only public schools and had fewer years of schooling had a higher likelihood of rarely or never consulting with dentists. These findings are consistent with national studies in Brazil [26, 29, 33, 34], and with international evidence showing persistent inequalities in access to healthcare based on socioeconomic position, particularly in Ibero‐American contexts such as Spain [3].

This apparently contrasting pattern may reflect the organization of the Brazilian health system, in which individuals with lower socioeconomic trajectories, often marked by exclusive attendance of public schools, tend to rely more heavily on SUS for medical care, particularly within primary healthcare services. In contrast, access to dental services in the public system remains more limited and irregular, which may disproportionately affect individuals with fewer socioeconomic resources and help explain the lower utilization of dental care observed in this group.

Although there has been improvement in the access to dental consultations in Brazil [26, 35], financial limitations, the lack of formal education, and the scarcity of public oral healthcare services are still significant challenges [29, 32, 33, 36]. Previous literature reports that Brazilians with higher levels of education had a higher frequency of dental consultations in the last 12 months when compared to those with no access to schooling or lower levels of education [13, 37]. Evidence from Ibero‐American contexts also shows that education and income strongly determine healthcare access, particularly in oral health [3, 9, 38].

These findings reinforce that financial barriers are a common determinant of delayed healthcare utilization across different health systems [9, 38]. In Brazil, seeking healthcare services is more common among those who have health insurance plans. Health insurance plans may facilitate access to services by mitigating potential financial barriers and reducing waiting times for treatment [30, 38]. In this study, the semiannual frequency of medical consultations among transgender individuals was more likely among those who solely used SUS. This finding suggests that SUS may play a protective role in reducing financial barriers.

On the other hand, in this study, SUS has not been able to overcome the barrier of unemployment, which is an obstacle for the transgender population to access oral healthcare. The association between unemployment and the difficulty to access healthcare services in Brazil is particularly relevant due to the high unemployment rate [4, 29, 38]. However, since unemployment can be a proxy for broader social exclusion processes, further studies are needed to disentangle these effects.

Brazilians reliant on SUS often experience greater social and structural vulnerability. They may face several barriers to access healthcare services, such as lack of transportation, lack of health information, and low availability of specialized services [29, 38]. The transgender population faces additional challenges. They experience high rates of unemployment and workplace discrimination, which results in greater dependence on SUS, greater exposure to social and structural disadvantage, and lack of access to oral health services [26, 31, 32, 33].

Gender‐based violence can lead to increased stress, which causes and exacerbates health problems [35, 36]. Among the participants of this study, individuals who had experienced gender‐based violence were more likely to rarely or never have dental check‐ups. This association does not imply causality but suggests that experiences of discrimination and violence may contribute to avoidance of health services, as reported in other studies from Brazil and other Ibero‐American countries [3, 10, 34, 38].

Previous literature highlights that experiences of institutional discrimination in healthcare services substantially increase the likelihood of avoiding medical consultations among transgender people [17, 36]. The inadequate training of healthcare professionals regarding the specific needs of the transgender population, combined with the refusal to use their social name and the lack of welcoming practices in public healthcare services, further reinforces their vulnerability [9, 10, 38].

This study highlights that the type of school attended, the number of years of schooling, unemployment and previous experience of gender violence might impair dental consultations by Brazilian transgender people. The Brazilian SUS, despite reducing financial barriers to medical care, has not been sufficient to ensure adequate access to dental services for transgender individuals. The intersection of discrimination, socioeconomic vulnerability, and the absence of effective public policies generates the urgent need for actions that promote comprehensive and humanized healthcare for this population [10].

Population‐based evidence on the health and healthcare utilization of transgender individuals remains limited, particularly regarding oral health. This study addresses an important knowledge gap by providing one of the largest national studies to simultaneously examine medical and dental healthcare utilization among transgender adults in Brazil, offering empirical evidence that has been largely absent from both national and international public health dentistry literature.

Taken together, these findings suggest that the patterns observed in this study are not isolated to the Brazilian context, but resonate with broader Ibero‐American evidence on health inequities affecting transgender populations. This paper provides original evidence by showing how educational attainment, type of school attended, unemployment, income, and experiences of violence shape healthcare utilization among Brazilian transgender individuals. These findings align with broader Ibero‐American research on health inequities, emphasizing the need for regional public health strategies that go beyond national policies and address structural determinants.

### 4.1 Limitations

This study has limitations that should be considered. The use of snowball sampling, although necessary to reach a hard‐to‐access population, may have introduced selection bias and limited representativeness, compounded by the lack of reliable official data on the size and distribution of transgender individuals in Brazil. Responses to the self‐administered electronic questionnaire may have been affected by social desirability bias, and no formal validation or pilot testing of the instrument was conducted. In addition, formal testing of some regression model assumptions, such as multicollinearity among sociodemographic variables, was not performed, which may affect the robustness of the estimates. The dichotomization of medical appointment utilization may have grouped individuals with no medical visits together with those with infrequent use, which represent distinct situations. This analytical choice was adopted to ensure sufficient statistical power and model stability and should be considered when interpreting the findings. Finally, the cross‐sectional design precludes causal inferences.

## 5 Conclusion

This survey showed that Brazilian transgender individuals predominantly used the public healthcare system, attended medical appointments semiannually, and rarely or never attended dental appointments. Attendance at public schools, lower educational attainment, unemployment, lack of income, and prior experiences of gender‐based violence were associated with lower use of health services, especially dental care. These findings reflect social and structural vulnerabilities that shape patterns of healthcare utilization rather than causal relationships. The results emphasize the importance of strengthening public policies that address socioeconomic inequalities, improve access to dental services, and promote inclusive and equitable healthcare for transgender populations.

## Funding

This work was supported by the Brazilian Ministry of Health (Ministério da Saúde do Brasil); the Brazilian Ministry of Women, Family, and Human Rights; the Dean of Postgraduate Studies at the Federal University of Minas Gerais (PRPQ/UFMG); the National Council for Scientific and Technological Development—CNPq (INCT Oral Health and Dentistry, grant no. 406840/2022‐9); and the Minas Gerais Research Support Foundation—FAPEMIG.

## Consent

Informed consent of all survey participants was received for their involvement in the surveys.

## Conflicts of Interest

The authors declare no conflicts of interest.

## Supporting information


**Data S1.** Checklist.

**Appendix A.** Study Questionnaire.

## Data Availability

The data that support the findings of this study are available from the corresponding author upon reasonable request.
