# Supplementary material for: Factors Associated With Health Care Utilization Among Transgender Adults in Brazil
Source: J Public Health Dent. 2026 Mar 3;86(2):193–203. doi: 10.1111/jphd.70050 (PMC13241909; doi:10.1111/jphd.70050)
Supplement: Supplementary file 2 — Appendix A. Study Questionnaire. [file JPHD-86-193-s002.pdf]

Appendix A

Study Questionnaire

TOPIC 1: PERSONAL IDENTIFICATION

1 – How old are you?

2 – In which municipality do you currently reside?

3 – According to the IBGE classification with regard to skin color, race/ethnicity, you identify yourself:

(__) white

(__) black

(__) brown

(__) yellow

(__) indigenous

4 – Which gender do you identify with:

(__) female

(__) male

(__) *others (leave field open)*

5 – Do you use your social name?

(__) Yes. I use it everywhere I go.

(__) No. I can't use the social name.

(__) I don't need to, because I have already modified my civil registration documents.

6 – Do you have children?

(__) Yes

(__) No

7 – What is your marital status?

(__) single

(__) married

(__) divorced

(__) widow(er)

(__) in a stable union

8 – With regard to religiosity, do you practice:

(__) Christianity

(__) Spiritism

(__) Buddhism

(__) Umbanda

(__) Candomblé

(__) Judaism

(__) atheism

(__) other:________________

TOPIC 2: EDUCATION AND PROFESSIONAL TRAINING

9 – What is your education?

(__) complete elementary school

(__) incomplete elementary school

(__) complete high school

(__) incomplete high school

(__) complete technical and vocational course

(__) incomplete technical and vocational course **(Open a second field to enter the course that was taken)**

(__) complete higher education

(__) incomplete higher education

(__) graduate studies

(__) *No schooling* **(In case of filling it out do not go to the next question)**

10 – Your education took place:

(__) totally in public schools

(__) entirely in private schools

(__) entirely in private schools with scholarship

(__) partially in public schools

(__) partially in private schools with scholarships

TOPIC 3: EMPLOYMENT AND INCOME

11 – Currently you are:

(__) formally employed (with a formal contract or agreement)

(__) informally employed (without a formal contract or agreement)

(__) Intern/scholarship holder

(__) Autonomous

(__) Retired due to disability

(__) Retired by time

(__) unemployed

Open box to put the time the person is in that situation, put time range (after each track)

12 – What is your monthly income?

(__) No income

(__) Up to R$ 500.00

(__) R$ 500 to R$ 1,000

(__) from R$1,001 to R$3,000

(__) from R$3,001 to R$5,000

(__) from R$5,001 to R$7,000

(__) from R$7,001 to R$10,000

(__) more than R$10,000

13 – Do you receive any financial aid from the government?

(__) Yes

(__) Bolsa Família Program

(__) Social Tariff for Electricity

(__) Housing allowance or rent voucher

(__) other ______________

(__) none

TOPIC 4: USE OF HEALTH SERVICES

14 – Do you have any chronic diseases?

(__) Diabetes

(__) Hypertension

(__) Asthma or other lung disease

(__) Kidney disease

(__) HIV/AIDS

(__) Other___________________________

15 – Do you have any Mental Health demands?

(__) Yes (open field to enter which one)

(__) No

16 – What type of health services do you use?

(__) Unified Health System – SUS

(__) Health Plan

(__) Particular

(__) None

17 – How often do you go to a routine doctor's appointment to find out about your health?

(__) Weekly.

(__) Monthly.

(__) Semi-annually.

(__) Annually.

(__) Greater than annually.

(__) Never

18 – How often do you see a dentist?

(__) Never.

(__) Rarely.

(__) In emergency situations

(__) Regularly.

19 – How do you find out about health?

(__) Through the press.

(__) By TV shows.

(__) On the internet.

(__) Through social networks (*WhatsApp*, *Facebook*, *Instagram*, others)

(__) With health professionals.

(__) With friends

TOPIC 5: SECURITY

20 – Have you ever suffered any violence because of your gender identity?

(__) Yes.

(__) No.
